# Unprofessional Conduct

**Published:** 1896-03

**Authors:** 


### Unprofessional Conduct.

What constitutes unprofessional or dishonorable conduct? is the question asked by the *Atlantic Medical Weekly*, which proceeds to answer as follows:

The words "unprofessional or dishonorable conduct" are declared to mean:

1. The procuring or abetting in procuring a criminal abortion.
2. The employment of "cappers or steerers."
3. The obtaining of any fee, or the assurance that a manifestly incurable disease can be permanently cured.
4. The willful betrayal of a professional secret.
5. All advertising of medical business in which untruthful and improbable statements are made.
6. All advertising of any medicines, or of any means, whereby the monthly period of women can be regulated, or the menses re-established, if suppressed.
7. The conviction of any offense involving moral turpitude.
8. Habitual intemperance.

If every person who recommends in an advertisement the use of any medicine which will do either of these things is considered as practicing medicine, the time is not far distant when the press will be purged of one of the most flagrant forms of quackery.—*St. Louis Med. and Surg. Journal.*

HARVARD Medical Faculty has decided that after June, 1901, candidates for admission to the medical school must present a degree in arts, literature, philosophy, science or medicine from a recognized college or scientific school.

